# Parents’ perspectives on supporting children during needle-related medical procedures

**DOI:** 10.3402/qhw.v9.23759

**Published:** 2014-07-08

**Authors:** Katarina Karlsson, Ann-Charlotte Dalheim Englund, Karin Enskär, Ingela Rydström

**Affiliations:** 1 School of Health Sciences, University of Borås, Borås, Sweden; 2 Department of Nursing Sciences, CHILD Research Group, School of Health Sciences, Jönköping University, Jönköping, Sweden

**Keywords:** Needle-related medical procedures, lived experience, caring science, younger children, phenomenology, reflective lifeworld research

## Abstract

When children endure needle-related medical procedures (NRMPs), different emotions arise for the child and his/her parents. Despite the parents’ own feelings, they have a key role in supporting their child through these procedures. The aim of this study is to describe the meanings of supporting children during NRMPs from the perspective of the parents. Twenty-one parents participated in this study. A reflective lifeworld research (RLR) approach was used and phenomenological analysis was applied. The essential meaning of the phenomenon—supporting children during an NRMP—is characterized as “keeping the child under the protection of one’s wings,” sometimes very close and sometimes a little further out under the wingtips. The essential meaning is additionally described through its constituents: paying attention to the child’s way of expressing itself, striving to maintain control, facilitating the child’s understanding, focusing the child’s attention, seeking additional support, and rewarding the child. The conclusion is that parents’ ability to be supportive can be affected when seeing their child undergo an NRMP. To regain the role as the child’s protector and to be able to keep the child “under the protection of one’s wings,” parents need support from the staff.

Parents experience various emotions when their child has to endure different medical procedures (Bernard & Cohen, 2006; Blount, Piira, & Cohen, 2003) and previous research shows that children also consider these procedures as distressing aspects of being sick and hospitalized (Lindeke, Nakai, & Johnson, 2006; Salmela, Aronen, & Salanterä, 2011; Salmela, Salanterä, & Aronen, 2009). Hospitalized children may undergo several needle-related medical procedures (NRMPs), for instance, intravenous cannulations, injections, capillary sticks, and venepuncture, which can cause different feelings for the child (Blount, Pirra, Cohen, & Cheng, 2006; Ellis, Sharp, Newhook, & Cohen, 2004; Young, 2005) but also for the child’s parents (Bernard & Cohen, 2006; Power, Liossi, & Franck, 2007).

This study was conducted in Sweden where the principle of child health care is based on parents’ attendance and involvement (c.f. European Association for Children in Hospital [EACH], 2006).

This is the second study of four in a larger project aimed at generating knowledge about the various aspects of how children experience and cope with NRMPs and the support that is provided for them. The perspectives of the child, the parents, and the nurses are included in this larger project. This article focuses on the meaning of supporting children during NRMPs from the perspective of the parents.

## Background

Parents have a role in acting as their child’s protectors (Bowlby, 1988), and when the child has to undergo medical procedures, the parents also experience different emotions (Bernard & Cohen, 2006; Power et al., 2007) that might affect their ability to protect their child. When children have to undergo an NRMP, is it important for them to have their parents present to help them to counteract their worries and fears (Power et al., 2007; Salmela et al., 2011; Salmela, Salanterä, Routsalainen, & Aronen, 2010) and being present is also something that most of the parents themselves prefer (Jones, Oazi, & Young, 2005).

All children will, at some time in their life, encounter different care environments and endure unpredictable situations (Blount et al., 2006; Power et al., 2007). What children perceive as unknown and as something that they do not understand may increase their fears and pain and may affect the perception that they have regarding the procedure (Salmela et al., 2011). An NRMP may be perceived as just such an action and may lead to different emotions surfacing in the child (Duff, 2003; Gaskell, Binns, Heyhoe, & Jackson, 2005; Melhuish & Payne, 2006; Meltzer et al., 2009) and in the parents (Bernard & Cohen, 2006; Power et al., 2007). Uman, Chambers, McGrath, and Kisely (2006) explain an NRMP as an investigation or action that children have to endure to prevent illness, to enable diagnosis and treatment, and that involves needles. Kortesluoma and Nikkonen (2004) and the review presented by Young (2005) highlight how some younger children have more negative experiences with regard to needles. The International Association for the Study of Pain presents a definition of pain as “an unpleasant sensory and emotional experience associated with actual or potential tissue damage or described in terms of such damages” (Loeser & Treede, 2008, p. 475). Several mechanisms are included in the experience of pain such as the sensitive (peripheral pain receptors), cognitive, affective and emotional mechanisms, and past experiences (Shankland, 2011). How children experience NRMPs results from a combination of these mechanisms, which are also based on the individual child, the type of procedure (Ortiz, López-Zarco, & Arreola-Bautista, 2012), and the supporting capacity of the parents (Taddio et al., 2010).

The staff also have an important role to play in alleviating children’s negative experiences due to NRMPs. Apart from providing the pharmacological treatment itself, the staff can support children during procedures by the use of distraction, hypnosis, and combined cognitive behavior therapy (Uman et al., 2006). Additional supportive measures by nurses are presented in a study by Karlsson, Rydström, Enskär, and Dalheim Englund (2014), which shows, among other things, that conversation is an important way of developing a relationship with children and their parents and is one way of supporting them during NRMPs.

Because parents’ presence is important during an NRMP, according to the children (Salmela et al., 2011), parents must be encouraged to support their child using different supportive measures. Distraction is a good tool to use during procedures and is described by McCarthy and Kleiber (2006) as those parents who help the child to focus on something other than the procedure, something positive, thus reducing the pain and distress that the procedure entails. Schechter et al. (2007) review shows that distraction has the best effect on younger children; that is, children under 7 years old. Salmon and Pereira (2002) also found that especially younger children might need help from their parents to make use of distraction. Additional supportive measures for children are different strategies such as “breathing techniques, relaxation techniques, books, games and puzzles, imagery and make believe, music and television, sensory experiences, and positive reinforcement” (Gaskell et al., 2005, p. 26).

Even though there is much research on parents’ supportive ability when children undergo NRMPs, there seems to be a gap in the literature concerning the meaning of supporting children during NRMPs from the perspective of the parents. The reason for this study is to add to the understanding of parents’ experiences of supporting their children alongside care development for the children. For a better understanding, is it thus necessary to add to the present knowledge with knowledge about the meaning of supporting children during NRMPs. The aim of this study is thus to describe the meanings of supporting children during NRMPs from the perspective of the parents.

## Method

### Design

The study has a descriptive design to illustrate the phenomenon of supporting children during NRMPs as it is lived and experienced by parents. A reflective lifeworld research (RLR) approach with phenomenological analysis was applied to illuminate the essential meaning of the phenomenon as well as its nuances (Dahlberg, Dahlberg, & Nyström, 2008). Ideas and thoughts from Husserl (1950/1977), Heidegger (1962/2008), and Merleau-Ponty (1945/2002) form the basis of lifeworld phenomenology and caring science. Scientific accuracy has been carefully considered, which meant having a phenomenological attitude throughout the study, and this consisted of using a critical and reflective approach, where openness, sensitivity, and bridling were prominent terms. Openness included listening to the parents with both sensitivity and awareness around what was being said; thus, listening humbly in order to be open to the other’s lifeworld and to capture the meaning of the phenomenon. Bridling meant not hurrying, enabling reflection, and letting the phenomenon show itself in its rarity (Dahlberg et al., 2008).

The study was conducted through video-recorded observations from the NRMPs, followed by face-to-face interviews and phone interviews.

### Settings

The study was conducted in the southwest of Sweden (with a population of 1.6 million people) at dissimilar units; namely, child health care services, pediatric primary care services, pediatric inpatient care, and pediatric outpatient care.

### Parents

Twenty-one parents participated in the study and were recruited by nurses. The inclusion criteria were that the parents would be able to understand and speak Swedish; that the parents, during the study period, participated in an NRMP with their child aged 3–7 years with a non-acute or life-threatening illness; that they were willing to participate; and that the child and nurses gave their informed consent. The selection of the parents was based on their child’s participation, which will be presented in an forthcoming article. Both the age of the parents as well as their occupation varied across the sample. The parents lived in rural as well as urban areas. On 14 of these occasions, the mothers were present; at 3, the fathers were present; and 4 times, both parents were present. On three of these occasions, siblings or other relatives were also present.

### Data collection

The data collection was conducted from the spring of 2011 to the summer of 2012, supported by 21 video recordings from the NRMPs, meaning-oriented face-to-face interviews, and phone interviews. The NRMPs included in the study involved skin tests for allergies, blood sampling (venous or capillary), intravenous cannula insertion, needle insertion into central venous ports, and injections into the joints. Topical anesthesia (except for skin tests for allergies and capillary blood sampling) and inhalation/sedation with N_2_/O_2_ (for those receiving injections into the joint and needle phobia) were the pharmacological aids that the children received, all according to the standard routines within each unit.

All parents were interviewed face-to-face together with their child directly after the procedure had been performed. Separating the children and their parents was difficult to do and was not something that the parents or children agreed to; however, this did not seem to influence the interviews. The face-to-face interviews began with an opening question: “Would you like to tell me about your experience when supporting your child during NRMPs?” Then, follow-up questions were asked such as, “Can you tell me more?” and “How do you mean?” These questions were designed to support the parents in their reflection and to help them to express variations in their experiences. The parents began by describing the specific procedure that had just taken place and then they talked more generally about their experiences of the phenomena: supporting children during NRMPs. To enhance their ability to reflect on what had happened during the NRMP, the video observation from the NRMP was shown to the parents as a stimulus. Thereafter, the video observations were not used in this work. A few days after the face-to-face interviews, 11 parents were interviewed for the second time by phone. Parental willingness to participate in phone interviews was decided on during the face-to-face interviews. Those parents who indicated that their child needed support afterwards were contacted a few days after the procedure. The purpose of the phone interview was to find out more about the phenomenon of supporting children during NRMPs. An MP3 player was used to record the face-to-face interviews and the phone interviews and the interviews were transcribed verbatim. The face-to-face interviews lasted for a mean of 39 min (range 17–67 min), the phone interviews for a mean of 14 min (range 3–30 min), and the video observations lasted for a mean of 11 min (range 4–30 min).

### Data analysis

Transcribed data from face-to-face interviews and phone interviews were analyzed using RLR with a phenomenological approach (Dahlberg et al., 2008). A constant movement when going from the whole, to the parts, then back to the whole characterizes the analysis. Other characteristics are to have an open reflective approach, an active dialogue with the text as well as a bridling attitude during the whole process. To become familiar with the text as a whole, to capture the phenomenon, the starting point was to read the interview transcripts several times until the text was perceived as being well known. When the text felt familiar, as a whole, the next phase started by dividing the text into meaning units, which means into parts. These units were analyzed in relation to meanings. When all meanings in the text were identified and described with a few words, similar meanings were brought together into clusters and described at a more abstract level. The clusters are not seen in the presentation of the findings. They form a temporal pattern of meaning that helps the researcher to find the essence of the phenomenon. The essence describes a new whole, the invariant meaning of the phenomenon; that is, supporting children during NRMPs. Finally, the more contextual nuances of the phenomenon were identified and described. In RLR, these nuances are called constituents, which are the variations of the essence and they can to some extent be overlapping.

### Ethical considerations

Ethical approval was obtained from the Regional Ethical Review Board of Gothenburg (Dnr 724-10). The Helsinki Declaration (2008) was followed. Before the study started, approval from the managers of each unit was obtained. The parents, who came to the unit because their child was in need of an NRMP, were recruited by nurses by asking them if they were willing to participate. If both the parents and the child showed interest, the first author gave additional information, orally and in writing, and then explained that the NRMP would be video recorded. The face-to-face interviews were conducted with the child present. This may have affected the parents’ ability to express themselves fully about the NRMP, but the child’s presence during the interviews did not seem to have any influence on the interviews.

## Findings

The essence, the most abstract level, is presented first, followed by its constituents—the more contextual nuances of the phenomenon that vary and that can be illustrated with quotations from the interviews.

The essential meaning of supporting children during NRMPs is described as “keeping the child under the protection of one’s wings,” sometimes very close and sometimes a little further out under the wingtips. It is about protecting the child by accompanying him or her through an unpredictable and unknown experience. This involves situations where the child’s needs and feelings must be met as well as the staff’s requests. Emotions are pushed aside in favor of the child’s need for security, which is prioritized by all of the people involved acting in an adult manner with control over their own powerlessness and their emotional expressions. Control can, however, only be related to things that are able to be influenced; play and talk is adapted to the child’s ability to understand and process such things. If the support is perceived as insufficient by the child, it is important to continue to consider the child’s perspective by “moving the protection a bit further out under the wingtips” and accepting help from, for instance, nurses, allowing them to use their professional experience to provide support. Varied and nuanced support maintains trust in the adult world. The intention is to give the child hope that when the procedure is finished, everything will feel good again.

The meaning of the phenomenon of supporting children during NRMPs consists of the following six constituents that describe the nuances of the phenomenon: paying attention to the child’s way of expressing itself, striving to maintain control, facilitating the child’s understanding, focusing the child’s attention, seeking additional support, and rewarding the child.

### Paying attention to the child’s way of expressing itself

Paying attention to the child’s way of expressing itself during an NRMP concerns interpreting the child’s needs and also listening to what the child says.

Paying attention to the child’s way of expressing itself during an NRMP involves taking into account the child’s personality, age, illness, and current condition. To protect and support the child, it is necessary to interpret the child’s body language and facial expressions to find out if the child, for example, is afraid. It is not always the needle that is the cause of anxiety: *Once the patch was gone, he could let his fears and anxieties go*. The parents also talk about different expressions where they see the protest as a healthy sign: *And at the same time, he also resists more, and that might well be seen as a small sign of health*. Such behavior evokes feelings of a positive nature. The time between different NRMPs determines the need for support. The parents are not in agreement on this matter, and some parents feel that it is good to have a longer interval between each procedure: *Then they have time to process it in a way and recover*, whereas another says: *And then we noticed when the intervals between each time started to become shorter, it became a little harder*.

Paying attention to what the child expresses about the procedure is essential. If the child’s illness is linked to an increase in pain, the child can ask its parents to seek health care, even if it means a related NRMP. That the child is capable of seeing this connection feels good for the parents. One parent says: *The child has said it herself when she had pain in the knees’ that ‘now I want to go and have that injection’*.

Interpreting what the child needs is not always possible and this may raise feelings of thoughtfulness: *He didn’t cry or anything, but then afterwards he said, ‘Mum, it did hurt more than I thought it would’*. Failing to interpret what the child needs is experienced as being unable to provide the necessary support. When the parents are not able to interpret their child correctly, they also become concerned about what they as parents pass on to their child: *It’s as though she was almost proud that she hadn’t cried. So I started thinking, have we given her the idea that one is not allowed to cry then?*

### Striving to maintain control

Striving to maintain control in order to protect and support the child during an NRMP is done by withholding one’s feelings of anxiety in relation to the procedure.

Striving to maintain control during the procedure is described by parents alongside how they talk to their child: *Being calm and thinking that this is perfectly normal … never talking about it as something that hurts or is uncomfortable … we don’t make any big deal of it*. Maintaining control means controlling one’s emotions and body language so that the child will not be affected in a negative way, which places high demands on the parents. However, there are differences in how this control is enacted, based on the parents’ past experiences and their then-current fears around the procedures.

Striving to maintain control involves having a calm attitude, even if it is difficult to see the child undergoing an NRMP: *It’s first afterwards that sometimes when it’s been scary that I might start thinking*. As a parent, you do not want to see your child suffer, and one parent describes it like this: *You become so powerless, he has to do it, it feels like you’re letting your own child* down. Another parent says: *You can compare it with you letting him go into a room with monsters*. If the purpose of the procedure is perceived as being justified, it reduces the parents’ negative feelings and they become calmer: *As long as I know that it is not just for the sake of the needle procedure*. Despite these feelings, parents’ strive to maintain control when supporting their child.

Being aware of their own body language, even if the parents have their own experiences to cope with, makes it hard for them to be there for their child: *Because I’m extremely scared of injections and needles. I’ve always concentrated on not transferring it to him …. I’ve concentrated on being relaxed*. If they do not manage to control their body language, it can result in feelings of insufficiency. At the same time, they point out that parents who have a fear of needles are the only ones that can understand the child and know what the child is going through.

It is sometimes not the actual needle procedure but the test results that give rise to feelings of worries that can affect the parents’ ability to maintain control. They may also be worried about what the NRMP will lead to for the child in the future: *It’s like the doctor said, ‘if he’s become afraid once, he’ll always be afraid of the needle stick’ …. That wasn’t particularly comforting*. If the staff are able to interpret the parents’ needs when they are trying to maintain control, it is appreciated by the parents in terms of them maintaining control: *I need a lot of feedback …. Although I am an expert on my child, I’m not an expert in these circumstances*.

Striving to maintain control becomes particularly difficult when no supportive action helps and restraint becomes necessary: *Then we had to get it in [the needle] and could just hold calmly but firmly*. If the child’s condition is such that the procedure cannot be cancelled, then it is better to hold the child, instead of extending the procedure. This can evoke contradictory feelings such as sadness and powerlessness but also guilt and anger at letting it happen, being in a life situation where they as parents need to face this situation. Restraining the child can leave the parents with a feeling of internal chaos. It is important in such situations to maintain control over one’s feelings and to interact with the staff for the restraint to be supportive: *It feels as though there’s no point in carrying on like that, as it will be the same story over and over again*. The support given is then described as follows: *I’ve got him in my arms … so that he knows that you’re holding him, and are there and can kiss him. That is the comfort I can give*.

### Facilitating the child’s understanding

Facilitating the child’s understanding concerns protecting and supporting the child by helping the child to understand the reasons for and the importance of the upcoming NRMP. This is done by talking to and playing with the child.

Before the NRMP, preparation is done at home by helping the child to understand what the procedure is about and why it is necessary. This gives rise to positive feelings for the parents in that they are able to do something for their child to ease the upcoming procedure. Facilitating understanding is done through talk and play and is based on the child’s past experiences and current needs. If the child has previous experiences from NRMPs, preparation is not seen as so important: *She’s done it many times. It was just to tell her that it should be done*. On the other hand, if the procedure is to be carried out for the first time, more preparation is necessary: *a new measure thus requires more time* and the child can play by performing the procedure on a stuffed animal or on the parents: *It seemed to him to be;* ‘okay then I stick you’ [parent]. Another parent explains it like this: *So I sat down and drew everything for him so he would understand*.

After the procedure, the use of talk and play is also important to facilitate the child’s understanding and simplify processing. If the child’s expectations are not lived up to, the need for talking increases and the unspoken responsibility of doing so is placed upon the parents by the staff. Not magnifying what the child has experienced, but at the same time, helping the child to take the chance to talk is important: *You provide the opportunity and if she wants to take it, then she can*. How to express themselves in the conversation is essential, and some words are avoided because of negative connotations: *I think when you say that the child has been good in relation to this then there will be tensions associated with it*. Another way to support the child is to confirm their experience by talking: *He would like it to be confirmed that it hurt and he was sad*. To facilitate the child’s understanding also involves helping the child to communicate with others, for example, through talking to children who have been in a similar situation. If the action is not perceived as difficult by the child, the parents can make use of the previous procedure, and by talking about it, they can support the child for the next occasion. Facilitating the child’s understanding after the procedure is perceived as doing something good for their child.

Playing is one way of supporting the child after the procedure and this can facilitate how the child understands and processes what has happened. The child can play hospital with his/her siblings and friends, and the play can be all about what the child himself/herself has experienced: *He has his puppets, he has his bag that he received from play specialists and stuff that he’s doing*. The play can also result in some negative reactions in the child: *But you notice with him that no, it just gets worse … then he feels bad and he starts to think about it again*. The parents are in the background, ready to support the child when he or she is playing.

### Focusing the child’s attention

Focusing the child’s attention during the NRMP is an ambiguous task and can be done by the use of distraction, by talking, and by playing with the child.

Supporting children by focusing their attention on something other than the procedure protects them. This can be done by talking and playing with them, for instance, by talking about nothing in particular, or by reading a book or play: *It can be about anything. As long as the child is safe*. This gives a feeling of having something to do during the procedure. Distraction techniques vary, but they should be based on the child’s needs, which are interpreted. For example, the child could have stuffed animals or other toys from home, and could play with them during the procedure. Focusing the child’s attention can also be passed over to the staff: *The play specialists have been there and blown soap bubbles and things like that*. To provide distraction is an established norm in the care of the children, but parents do not always feel that it is the best way to support their child. It helps to look at the implementation of the action, instead of placing a book in front of the child’s face to hide the procedure: *She is so curious; it can be more hush, hush if she cannot see*. The child who wants to see what happens is usually a child who has a need for control: *He wants to have control over everything … we have tried so that he doesn’t have to watch all the time, but no, he wants to watch*. Parents can feel regret when they allow the child to be distracted by staff rather than watching the procedure.

### Seeking additional support

Seeking additional support to protect and support the child during an NRMP is a twofold experience. This involves positive and negative feelings when working together with the staff and with each other.

Seeking additional support means acting as the child’s representative in order to protect the child and this can manifest itself in one way when handing over the responsibility for the support to the staff while remaining in the background: *Then you go with the flow*. This evokes feelings of insecurity but also of relief, as someone else has taken over the responsibility for a while. They are partly in the hands of the staff, and the parents, in the presence of the child, do as the staff ask, but only to a certain extent: *As a parent one should never doubt the nurses … and tell the children that ‘this is the way we are going to do it and now you have to listen’*. Seeking additional support from the staff is only done if the parents feel confident that the staff understand how much previous life events affect the child, because this also affects how the child will cope with the NRMP. Consequently, the staff must be able to put the child in focus, interpret what the child expresses and needs, make “small talk,” and continuously talk to the child about what they are going to do and what is happening.

If the staff do not have the time to put the child in focus, then the staff tend “just to perform” the procedure: *Then it’s often stressful and they just want to get it done*. Not giving the child enough time leads to the child needing more support from the parents afterwards: *They treated her as though she still was used to it … but she wasn’t … maybe she needed a little more time for preparation, information*. This causes a certain level of irritation among the parents.

The staff’s ability to support the child is closely monitored by parents through their senses, and if the support does not work, the parents take a step forward and take over: *If I have to do something then of course I’ll do it*, or as one parent describes: *We went in and we sat down and I said ‘let’s get it over fast’ . She walked over and then just did it …. If it is any one of those morose types who don’t know a thing about children and just goes on, then you have to intervene a little*. This means reclaiming the control over the supportive function.

Seeking additional support also occurs between the parents when supporting the child. If one parent experiences difficulties attending an NRMP, the other parent takes over; thus, the parents feel that one of them must be present during the procedure. If both parents find it difficult, it is usually the father who is involved, and, as one mother says: *He looks away, but he holds tightly or holds his hands*.

### Rewarding the child

Rewarding the child as compensation is one way of supporting the child at an NRMP and entails doing things together in relation to the procedure. The child receives some sort of comfort and compensation.

Supporting the child by rewarding him or her evokes a feeling of doing something encouraging as vindication for the child’s experiences. This can happen when the child is waiting for the procedure to be carried out in the hospital or when at home: *We usually play games on the computer together … snuggle on the couch and the child may choose some movies and stuff afterwards*. Another rewarding activity that the child appreciates is visiting the play department with its parents: *He has not been in preschool for a long time and played so that is something fun to go to, the play department*. The child gets to choose what suits him or her best: *You can’t force them to sit and play with beads every time … he must surely be allowed to do what he wants*. Rewarding the child can also mean that the child receives gifts, or as one parent says: *He gets treats and we go to McDonalds afterwards*. The children learn that after the procedure is over, they get gifts or can do something fun together with their parents. The children may perceive gifts as a sign that they have put up with something unpleasant. It is not always the gift that is the most important thing—it can be something completely different such as a colorful sticker: *She is more fascinated by a funny sticker or something like that*. If the child does not want to be part of the NRMP, gifts do not help, and other supportive measures need to be tested. Doing things together that are fun is important; otherwise, the NRMP becomes unbearable for the child.

## Reflections on the findings

This study was undertaken to describe the meanings of supporting children during NRMPs from the perspective of the parents. The analysis resulted in the following constituents: paying attention to the child’s way of expressing itself, striving to maintain control, facilitating the child’s understanding, focusing the child’s attention, seeking additional support and, rewarding the child. One of the surprising findings is that parents, under certain circumstances, consider restraint to be supportive. Additionally, in daily life, parents are used to having an embodied knowledge and an ability to comprehend the world just as their child sees it. However, during these medical procedures, these abilities may be affected, and that the parents themselves are thoughtful over their lost abilities is somewhat surprising.

To be a parent means that one has a role in protecting and caring for one’s child throughout its childhood. The parents’ role as protectors and their supporting abilities are essential throughout this study and are exemplified by “keeping the child under the protection of one’s wings,” sometimes very close and sometimes a little further out under the wingtips. This can be compared to Bowlby (1988), who states that during childhood, children need an adult who gives them a secure base or safe space to come back to when the child is exploring the world. The parents as protectors is something that the children also consider as important during hospitalization and is described in a study by Darcy, Knutsson, Huus, and Enskar (2014). From a nursing context, this also been discussed, in that parents should be enabled when it comes to acting in a manner that causes them to be able to maintain their role as the child’s normal protectors (McGrath, Forrester, Fox-Young, & Huff, 2002; Pearch, 2005: Schechter et al., 2007).

The parents in this study argued that they had to pay attention to their children’s way of expressing themselves to find out why their children were afraid and worried. This was done by parents interpreting their child, and if they did not interpret this correctly, the parents became thoughtful. Previous research from Cavender, Goff, Hollon, and Guzzetta (2004) shows that parents have an advantage because they already have an established relationship with their child, and that this means that they best know how to support their child. According to the findings from the present study, it seems that parents are not always capable of interpreting their child’s expressions. It may depend on the parents’ own feelings of worry and of being exposed. In these situations, the staff need to help the parents to feel secure with the intention of supporting the parents and thereby the child.

Findings from this study also indicate that parents strive to maintain control to protect their child and enable support. To do so, parents let the child know, verbally as well as with their body language, that they are available for the child during the whole procedure. Parents try to withhold their own anxiety and fear to protect the child. This was especially important when the child was restrained during NRMPs and the parents had to struggle with feelings of a negative nature. Previous research shows that parental pain expression can lower children’s pain threshold (Goodman & McGrath, 2003; Salmon & Pereira, 2002) and that parental behavior has a strong correlation with children’s level of distress, compared to the staff (Mahoney, Ayers, & Seddon, 2010). How parents communicate with their child during a distraction affects children (Salmon & Pereira, 2002; Vance & Eiser, 2004), and verbal expressions, for example, humor, distraction, and non-procedural talk reduce distress, and criticism, apologizing, and parental reassurance raise children’s distress during procedures (Mahoney et al., 2010).

Furthermore, during some situations, such as when restraint is carried out, an adult perspective of what is best for the child is used. This evokes thoughts about how the child’s perspective could be made visible. We believe that parents need to be reinforced so that they can maintain control and protect their child. This can be done with support from the staff through information, training parents in supporting actions, and by helping parents become more active during NRMPs. It is reasonable to assume that this will strengthen the parents and also the child.

The parents in this study specified that one job they could do to support their child was to focus their attention on something other than the NRMP by distracting the child. The parents felt that distraction was not always the best way to support their child and that this depended on the child’s need for control. The parents indicated that the staff could also distract the children. This is in line with a study by Karlsson et al. (2014), where the nurse’s supporting tasks were to distract the children, but also to interact with the parents to help them to distract their child. Previous research shows that it is likely that parents are less worried when a distraction is given because it helps children to be relaxed and also affects parents in a positive way in not seeing their child suffer. If the parents are the ones performing the distraction, they may also feel good about having a job to do (Dahlquist et al., 2002). To achieve a positive result, parents have to learn how to use parental-led distraction, even if it is not considered the same as distraction led by the staff when it comes to a decrease in pain (Taddio et al., 2010). The use of distraction gives children an opportunity to deal with the procedure and to counteract pain, worry, and anxiety (Cohen, 2008) and it has also been found to reduce children’s self-reported pain (Uman et al., 2006). To use different methods of distraction can be an advantage, as some children respond better to one form than another (McCarthy et al., 2010).

The use of distraction as a supporting action is not always beneficial, as demonstrated in a study by Tak and van Bon (2006). There was no evidence that distraction had a positive effect; actually, it appeared that distraction could increase children’s pain experiences, but the result was not significant. Similar findings are presented by McCarthy and Kleiber (2006), in that not all children find distraction helpful, and that not all parents are capable of supporting their children through distraction. Consequently, distraction can be both good and bad, and it is reasonable to assume that the staff must be aware if the child wants to be distracted and they also need to be aware of the parents’ ability to distract the child.

According to the parents in this study, they accepted help for additional support when they themselves could not fully support their child during NRMPs. The additional support could be from the staff or from the other parent. The parents’ conduct demonstrated that one of them had to be present at the NRMP. The importance of parental presence was revealed in a study by Karlsson et al. (2014), where the parents were the child’s base of security, according to the nurses. Piira, Sugiura, Champion, Donelly and Coles’s (2005) review highlights how many parents wish to stay and comfort their children during procedures and that parental distress did not increase when doing so, nor was there evidence that they were less satisfied. This is in alignment with a review by Boudreaux, Francis, and Loyacano (2002), and Bernard and Cohen (2006) further develop this in that the presence of parents and how it affects children’s (infants) levels of anxiety and pain most likely has to do with the parents’ own levels of anxiety. That parental presence is to the children’s advantage is presented in studies by Salmela et al. (2009, 2011), where children state that parents can be a good coping strategy for managing the procedure. We regarded it as obvious that parents felt the need to be a secure base for their child and that the child had a need for its parents as such a secure base during procedures. Not being able to or have the opportunity to attend an NRMP could hinder the parents’ protecting ability. Based on this, it is important to support parents to be present and to help them to support their child, based on the parents’ ability.

## Methodological issues

By using RLR and a phenomenological analysis (Dahlberg et al., 2008), a deeper understanding has been revealed of the phenomenon of supporting children during NRMPs from the perspective of parents.

The interviews with the parents took place with the child present and sometimes with siblings or a relative. The parents wanted their child to remain, and the child also wanted to stay, which probably had to do with the parents being the child’s base of security. If both parents were present, they both participated in the interview as long as the child attended. If the child was familiar with the unit, he or she sometimes went to the playroom for a while when the parents’ interview continued. The interview situations were sometimes interrupted by the staff when something had to be done with the child. This could be perceived as stressful for the parents and for the person carrying out the interviews. All of this together led to the interview situation sometimes being perceived as cluttered, but this seemed, under the circumstances, to be the only alternative. The care of children concerns not only the child, but also the whole family, which also applies in the research context. That the interviews were perceived as cluttered was handled by the first author focusing on the interview situation with an awareness of the parents’ experiences, listening actively, and having a genuine desire to see, hear, and understand the meaning of the phenomenon as experienced by the parents.

The researchers had a pre-understanding of the issue under study. The first and third authors are pediatric nurses and the second and last author have carried out research with children and parents. The pre-understanding was handled by discussing issues in relation to having a bridling attitude (c.f. Dahlberg et al., 2008). This includes writing down one’s thoughts about the phenomenon, and continuously discussing these thoughts among the co-authors and at seminars. It was also important not to reach an understanding too quickly, to be allowed to see the new, which is described by Dahlberg and Dahlberg (2003) as not taking “the indefinite as definite.” This was done by remaining as long as possible in the first step of the analysis; that is, going back and forth in the text in order to avoid misunderstandings due to preconceptions and ensuring that it was not the pre-understanding (Gadamer, 1960/2004) that the research provided answers to.

The findings are valid for parents from Sweden supporting children aged 3–7 years undergoing an NRMP, but can probably apply to children of other ages and in other cultures.

Hopefully, knowledge from this study may lead to a greater understanding of supporting children involved in NRMPs from the perspective of parents and this knowledge will help the staff in the care development of children undergoing NRMPs.

## Conclusion and clinical implications

For parents, seeing their child undergo an NRMP may affect them in many ways, and this may affect their ability to protect and support the child when having a procedure performed. To support their child, parents pay attention to the child’s way of expressing itself in relation to the procedure. The parents strive to maintain control by attempting to put their own worries and anxieties aside. They also try to put their child’s needs before their own needs and, to do so, parents often need support from the staff. Play and talk are used before the procedure to facilitate the child’s understanding by focusing the child’s attention; or afterwards, to help the child to process the event. Parents also felt that doing fun things together was vital in helping the child to manage undergoing the procedure.

Parents’ meanings around supporting their children during NRMPs must be clarified, which can be done through research. The staff are required to interpret the parents’ expressions, but they should also ask the parents for their opinions regarding, for example, how much responsibility they may wish to have during the procedure. This must be implemented in a tactful manner so that the parents really feel that they do have a choice. Learning how to do this could be done through tutorials in the workplace. Asking questions is a fairly obvious way to receive and to give information, but is also a good way to get the parents involved in the procedure. It is also important to encourage parents to ask questions and to share their experiences. For this to happen, parents need to feel welcome to do so, and the staff must counteract the stress that can easily build up when there is insufficient time available.

Giving information to the parents in order for them to increase their knowledge can be done through an information brochure, by phoning the parents a few days before the procedure, and by providing information or a link to a website that explains the action. It is also important to give information about why the procedure needs to be implemented; thus, it reduces the parents’ negative feelings and they become calmer. The parents’ ability to provide support can be affected, and it is important to inform the parents that this is a perfectly normal reaction to the procedure in order to counteract any negative emotions. Acting calmly and confidently, telling the child what will happen and asking the parents about their child are further important steps to make the parents feel comfortable in accepting help from the staff.

From a caring-science perspective, the present study emphasizes that to support children during NRMPs, it is important that the staff are aware of how parents can be affected by the process and of how the parents’ supportive capacity may become affected. Based on this, it is one of the staff’s most important tasks to support parents to enable them to support their child during NRMPs so that they can reclaim their role as the child’s protectors and keep the child “under the protection of their wings.”
